# Structural Basis for Monoclonal Antibody Therapy for Transthyretin Amyloidosis

**DOI:** 10.3390/ph17091225

**Published:** 2024-09-17

**Authors:** Avi Chakrabartty

**Affiliations:** 1 Department of Medical Biophysics, University of Toronto, Toronto, ON M5G 2M9, Canada; avijit.chakrabartty@utoronto.ca; 2 Proteotoxicity Solutions, Toronto, ON L4K 2E1, Canada

**Keywords:** transthyretin, amyloidosis, antibody therapy, protein structure, cryo-electron microscopy

## Abstract

The disease of transthyretin (TTR) amyloidosis (ATTR) has been known since the 1960s, and during the past 60 or so years, there has been a sustained period of steady discoveries that have led to the current model of ATTR pathogenesis. More recent research has achieved major advances in both diagnostics and therapeutics for ATTR, which are having a significant impact on ATTR patients today. Aiding these recent achievements has been the remarkable ability of cryo-electron microscopy (EM) to determine high-resolution structures of amyloid fibrils obtained from individual patients. Here, we will examine the cryo-EM structures of transthyretin amyloid fibrils to explore the structural basis of the two monoclonal antibody therapies for ATTR that are in clinical trials, ALXN-2220 and Coramitug, as well as to point out potential applications of this approach to other systemic amyloid diseases.

## 1. Introduction

Transthyretin amyloidosis is a protein misfolding disease with both sporadic and hereditary forms. In this disease, TTR, the serum transport protein for thyroxine and vitamin A, misfolds and is deposited as amyloid in peripheral nerves, causing polyneuropathy (ATTR-PN) in the heart, causing cardiomyopathy (ATTR-CM), and sometimes in other organs like the kidney, digestive tract, and eyes, causing various organ dysfunctions. Recent advances in the diagnosis and treatment of ATTR, which began in 2011 with the approval of Tafamidis [1], are having a significant impact on patients today. Diagnostic successes include new minimally invasive imaging technologies that precisely locate and measure the amyloid load in the patient [2]. Treatment successes involve the generation of three new types of therapy: silencers, stabilizers, and depleters [3,4]. Silencers reduce the expression of TTR protein through either gene-editing technology or RNA-based therapy, and consequently exhaust the supply of TTR molecules available to form amyloid. Stabilizers are small molecules that bind and stabilize the native tetramers of TTR, thereby diverting TTR molecules away from the misfolding pathway. Depleters are usually antibodies that remove amyloid fibrils, the final product of the misfolding pathway.

While both silencer and stabilizer types of drugs have been approved by the FDA, the silencer drugs have the disadvantage of requiring vitamin A therapy, as TTR expression is inhibited by these drugs, leading to faulty vitamin A transport [5,6,7]. Stabilizers do not affect the vitamin A binding site on TTR, but they do affect the thyroxine binding site, where the stabilizer drug competes with the natural ligand, thyroxine. Fortunately, TTR is a minor carrier of thyroxine, and stabilizers appear not to affect thyroid function [8,9]. Only two monoclonal antibody drugs have been evaluated in clinical trials: ALXN-2220 and Coramitug (Table 1). It is too early to comment on the disadvantages of this type of therapy. These two monoclonal drugs as well as related antibodies that did not make it to the clinical trial stage will be the major focus of this article.

**Figure 1:**
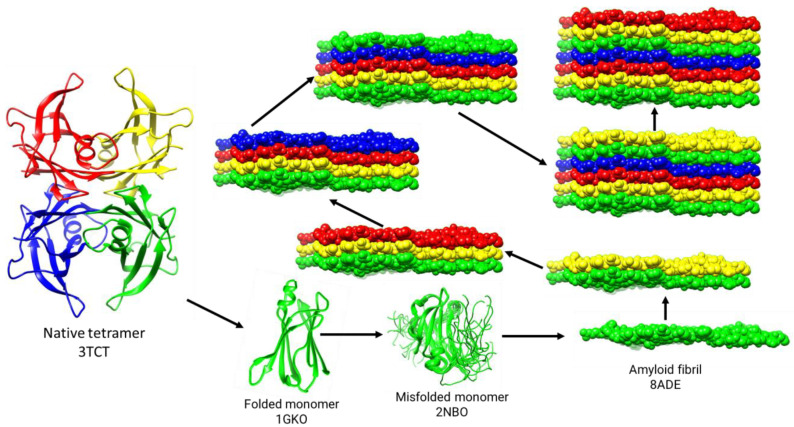
Based on experimental findings, a proposed pathway for the misfolding of TTR is presented. The Protein Data Bank (PDB) codes are provided for each class of structure shown. The native tetrameric TTR (3TCT), when subjected to mutation and/or exposed to environmental stressors, becomes unstable and dissociates into monomers (1GKO). The overall instability of the isolated monomers causes conformational fluctuations and misfolding (2NBO), resulting in the transient formation of a flattened stackable monomer (8ADE). The spontaneous stacking of these flattened monomers as they are formed is energetically favorable and leads to the growth of amyloid fibrils. Protein structures were rendered using Chimera 1.13.1.

The TTR misfolding pathway [22], elucidated by a combination of X-ray crystallography, NMR spectroscopy, cryo-EM, and various biochemical techniques, describes a plausible process by which genetic and/or environmental factors destabilize the native tetrameric structure of TTR, causing the molecule to dissociate into monomers. The unstable nature of the isolated TTR monomers induces conformational fluctuations that result in the transient formation of a flattened monomer that stacks with other flattened monomers as they form. This flattened stackable monomer represents the growth unit of the amyloid fibril. The continued growth of the amyloid fibril is believed to be a spontaneous downhill polymerization process [22]. This pathway is illustrated in Figure 1. This pathway is meant to be conceptual and is not a complete depiction of the process; it introduces the players of the misfolding process.

Early experiments, which were crucial for establishing the misfolding pathway, showed that the incubation of TTR at pH 3.6 to 4.8 was all that was required to initiate amyloid fibril formation [23,24]. Surprisingly, the mild acidic incubation that produced amyloid fibrils also caused the appearance of monomers and the disappearance of tetramers. This led to the conclusion that TTR might need to dissociate into monomers before forming amyloid fibrils, thus establishing the monomer as a key misfolding intermediate [23,24].

It was later found that ATTR-causing mutants of TTR dissociated into monomers more readily than the wild type when exposed to mildly acidic conditions. This difference raised the possibility that ATTR mutations cause disease by destabilizing the tetramer relative to the monomer [25,26]. Studies using an engineered monomeric mutant of TTR demonstrated that the engineered monomer forms a well-folded structure at neutral pH that is nearly identical to that of the monomeric subunit in the native tetramer [27] (Figure 1; pdb code 1GKO). At acidic pH, however, the engineered monomer forms amyloid fibrils at a much faster rate than tetrameric TTR [22], implicating the dissociation of the TTR tetramer as the rate-limiting step of the TTR misfolding pathway [22].

The engineered monomer still exhibits the same preference for slightly acidic aggregation conditions as wild-type tetrameric TTR, suggesting that the monomer converts from a relatively stable conformation at neutral pH to a misfolded monomer at slightly acidic pH [27]. Amide proton exchange experiments have provided evidence that in this acid pH range, specific subdomains comprising several residues in β-strands C, B, E, and F are partially unfolded [28]. The solution structure of the engineered monomer under conditions close to aggregation [24] (Figure 1; pdb code 2NBO) reveals significant partial unfolding. The structure of this partially unfolded engineered monomer is likely to be very similar to the misfolded monomer postulated to be transiently populated during amyloid fibrillogenesis [28,29].

Cryo-EM has provided the highest resolution structures of the conformational states of amyloid fibrils, the final product of the TTR misfolding pathway (Figure 1; pdb code 8ADE). Currently, there are over a dozen cryo-EM structures of TTR amyloid fibrils in the Protein Data Bank. The structures of these amyloid fibrils will be discussed in detail in a later section.

## 2. Mechanisms of Action of Silencers, Stabilizers, and Depleters

Available and emerging therapies are all focused on targeting the misfolding pathway of TTR (Figure 1). These therapies include silencers such as CRISPR/Cas9 editing the TTR gene [4,30] and targeting TTR mRNA with small interfering RNAs [31] and antisense oligonucleotides [32]. Silencers work by significantly reducing the concentration of TTR tetramers in the blood through nucleic acid-based therapeutics. Through eliminating the tetramers, the formation of monomers is prevented, which in turn stops further fibril growth. When it comes to amyloid fibrils that were deposited prior to silencer treatment, the clinical trial data of the silencer Patisiran show a clear reduction, but not elimination, in cardiac amyloid over a one- to three-year period [4,33]. Therefore, it seems that silencers do not completely remove amyloid that was deposited before treatment started.

The development of the stabilizer drug Tafamidis [3] began with the discovery that thyroxine, the natural ligand of TTR, binds to the TTR tetramer and inhibits amyloid fibril formation [34]. This discovery led to the realization that the unnatural ligand flufenamic acid also binds to the thyroxine binding sites of TTR and inhibits amyloid fibril formation [35]. As a result, targeted high-throughput screening efforts were initiated to find high-affinity TTR tetramer binders [36]. These efforts ultimately led to the development of Tafamidis by Kelly and coworkers [37], which became the first approved treatment for ATTR [1]. The mechanism of action of Tafamidis is to offer an alternative to the misfolding pathway, where the tetramer can become more stable by binding a stabilizing ligand like Tafamidis, thus avoiding misfolding. The effectiveness of Tafamidis has been demonstrated in the ATTR-ACT clinical trial, which showed that Tafamidis treatment was associated with lower all-cause mortality, a lower rate of cardiovascular-related hospitalizations, and a lower rate of decline in tests of exercise tolerance (6MWT) and health status assessments among heart failure patients (KCCQ-OS) [38]. However, like silencers, stabilizers target the tetramer and do not seem to greatly affect the amyloid that was deposited before treatment was started. This inability to remove previously deposited amyloid may explain the clinical results showing that Tafamidis slows down disease progression, but immediate improvements in symptoms have not been observed.

Certain depleter antibodies can also function as fibril inhibitors. These antibodies bind to monomers and fibrils, but not to native tetramers. In this situation, the antibody itself serves as a fibril inhibitor by binding to monomers. The immune complex that forms between the antibody and the monomer is unable to contribute to fibril growth, thus preventing it. Depleter antibodies also bind to amyloid fibrils. If they bind to the ends of the fibril, they may interfere with the polymerization interface and effectively seal off the fibril, preventing further growth.

However, the most significant effect of the antibody is the removal of antibody-tagged monomers and fibrils by macrophages and the immune system. Therefore, unlike silencers and stabilizers, these antibodies have the potential for dual action in reducing fibril deposits. First, they act as fibril inhibitors, and second, they facilitate the immune clearance of monomers and amyloid fibrils. This dual mechanism of action sets depleter antibodies apart from silencers and stabilizers. The binding constants for stabilizers and depleters are compared in Table 2. Interestingly, both stabilizers and depleters possess moderate nanomolar affinities (Table 2). Below, we will provide a detailed description of the development of depleter antibodies.

## 3. Antibodies to TTR and Their Binding Specificities

Antibodies to TTR have been commercially available since the mid-1960s when TTR (originally called prealbumin) was discovered [42,43]. These early antibodies were used in immunoelectrophoresis experiments to demonstrate that TTR is a vitamin A transport protein [44]. They were also used in immunoadsorption experiments to demonstrate that TTR is a thyroxine transport protein [45]. In a classic paper in 1978, it was shown that antibodies raised against amyloid fibrils isolated from patients with familial amyloid polyneuropathy (now known as ATTR-PN) cross-reacted with purified TTR protein [46]. Reciprocally, commercial antibodies raised against purified TTR protein cross-reacted with amyloid fibrils from patients, thus establishing that the amyloid deposits from ATTR-PN patients are composed of TTR [46]. The immunohistochemical demonstration that the cardiac amyloid deposits observed in ATTR-CM were also composed of TTR was achieved shortly afterward in 1981 [47].

Antibodies have also been used to identify regions of TTR that are exposed in the amyloid fibril versus regions that are exposed in the native tetramer. In 1994, Westermark and coworkers [48] used a collection of antibodies polyclonal to TTR peptide fragments, TTR amyloid fibrils, and TTR tetramers to map amino acid exposure in fibrils and tetramers. They found antibodies directed to residues 24–35, 50–60, and 90–100 bound both TTR tetramers and amyloid fibrils, while antibodies directed to residues 115–124 bound specifically to TTR amyloid fibrils. An examination of the TTR tetramer structure has shown that both residue segments 24–35 and 50–60 are widely exposed in the tetramer and should be available for antibody binding. However, the 90–100 residue segment is buried in the native tetramer and should not be able to bind to the antibody. Cross-reactivity or nonspecific binding with other antigenic components in the sample may explain why antibodies to TTR (90–100) appear to bind native TTR tetramers. The antibody to residues 115–124 does appear to bind specifically to amyloid fibrils but not native tetramers, which is consistent with this epitope being significantly buried in the native tetramer. By implication, this epitope, residues 115–124, should be exposed in the amyloid fibril structure.

An interesting study produced two monoclonal antibodies that specifically bound to amyloid fibrils, but not to native wild-type tetramers [17]. That study used an unnatural, highly amyloidogenic mutant of TTR as an antigen [44]. The hybridoma screening test identified two different monoclonals that showed specific binding to fibrils, but not tetramers. The epitope mapping of the two monoclonals indicated that one monoclonal specifically binds residues 39–44, while the other binds residues 56–61. The anti-TTR (39–44) antibody has been shown to be a useful clinical laboratory tool. It produces a positive immunoassay signal in blood plasma samples from ATTR-PN patients and asymptomatic carriers, but no signal from patients who do not have any mutations in TTR or have nonamyloidogenic mutations [18]. The anti-TTR (39–44) antibody immunoassay signal from plasma has high potential to be a biomarker or diagnostic for ATTR. The anti-TTR (39–44) has also proved to be a useful research tool for investigating the amyloid fibril formation of TTR variants [19,20]. Other antibodies specific to TTR amyloid fibrils have also been produced using amyloid fibrils generated from recombinant TTR and TTR mutants as antigens [21]. The epitopes of these TTR fibril-specific antibodies have not been determined.

Ando and coworkers developed RT24 [16], which is a humanized monoclonal form of the polyclonal anti-TTR (115–124) described above [48]. RT24 has been shown to bind to TTR fibrils but not tetramers. More importantly, RT24 has been shown to inhibit fibril formation [16]. Additionally, RT24 has been shown to form immune complexes with fibrillar TTR that can be readily phagocytosed by human macrophages [16]. RT24 could be one of those potential dual-action therapeutic antibodies discussed earlier, which act through both fibril inhibitor action and immune clearance of amyloid.

Humans sometimes produce antibodies against their own TTR. Unique IgM molecules (catabodies), which were isolated from pools of blood serum obtained from healthy individuals with no history of amyloidosis, were found to hydrolyze TTR fibrils but not TTR tetramers [49]. The hypothesis is that a certain level of TTR misfolding occurs naturally in all individuals, and the immune system generates IgM catabodies to break down the misfolded TTR. No stable immune complexes form between the misfolded TTR and the IgM catabodies. This may be due to the spontaneous hydrolysis of the misfolded TTR molecules while bound to the IgM catabody.

The apparent stochastic generation of antibodies that aid in the clearance of amyloid has been observed in three different ATTR-CM patients [50]. These three male patients, aged 68, 76, and 82 years old, all had heart failure caused by ATTR-CM, but they had not yet received any disease-modifying treatments. Remarkably, during the routine management of these patients, and before any ATTR-specific treatments were initiated, a reversion to near-normal cardiac structure and function was observed. High titers of polyclonal IgG antibodies to TTR were detected in the serum of each patient. These antibodies were shown to bind to ATTR amyloid deposits and synthetic TTR fibrils. The clinical significance of these antibodies is being investigated. But most importantly, these findings show that ATTR in these cases is reversible.

## 4. ATTR Depleter Antibodies in Clinical Trials

**ALXN-2220 (NI006, NI301A, anti-TTR (41–45))** Neuroimmune AG, a company from Schlieren/Zurich, Switzerland, has also taken advantage of the fact that some humans produce antibodies against their own TTR [10,11]. They screened a human memory B cell library generated from healthy elderly adults to identify expressed antibodies that bind to TTR fibrils but not TTR tetramers. The antibody identified from this screening, NI006, binds to fibrils made from both wild-type and mutant TTR with nanomolar affinity, but does not bind to tetramers made of the same proteins. The epitope mapping of NI006 revealed that this antibody binds to residues 41–45 of TTR, which are partially buried in the TTR tetramer [11]. The epitope of NI006 overlaps with the epitope of anti-TTR (39–44), a previously developed monoclonal antibody mentioned earlier [17]. Both NI006 and anti-TTR (39–44) bind to TTR monomers, dimers, oligomers, and large aggregates; however, there are some differences in the extent of binding to monomers and large aggregates [11]. NI006 stains amyloid deposits in the heart and other tissues of both wild-type cases and several hereditary ATTR cases [21]. Immune complexes formed between NI006 and fibrils, composed of the aggregation-prone L55P mutant of TTR, were readily phagocytosed by cultured macrophages. When these immune complexes were added to patient myocardial tissue sections, they were taken up by resident macrophages in the patient tissue sample. NI006 was shown to aid in the clearance of amyloid material in a non-transgenic mouse model of ATTR [11]. In that experiment, amyloid material from an ATTR patient was injected into the thigh of a mouse, which was then injected with varying amounts of fluorescent NI006 antibody. The administered antibody caused an initial large increase in fluorescence at the injection site followed by a steady decrease, indicating that the antibody is binding to the injected material and facilitating its removal.

Based on these positive laboratory results, the company sponsored a phase 1 trial of antibody NI006 for the depletion of cardiac transthyretin amyloid [12]. This clinical trial demonstrated that NI006 met all the safety requirements for a therapeutic IgG antibody. But more importantly, cardiac imaging with bisphosphonate scintigraphy clearly shows a visible reduction in amyloid load with NI006 treatment [12]. Cardiac MRI measures of extracellular volume, which is an index of cardiac amyloid load, also showed significant decreases with NI006 treatment. Cardiac biomarker levels did show a reduction with NI006 treatment at the 12-month mark of the study, indicating a degree of healing. With the fortunate circumstance of having shown efficacy at the phase 1 level, we all wait in anticipation for further clinical trial results. ALXN-2220 is the new designation for NI006, and Alexion-AstraZeneca will be sponsoring the next clinical trial.

**Coramitug (PRX004, anti-TTR (89–97))** This depleter antibody started out as a polyclonal antibody developed in this author’s academic lab at the University of Toronto [13]. The epitope (TTR 89–97) was selected through a structure-based approach where the structures of tetrameric TTR and monomeric TTR were compared segment by segment. Five to ten continuous residue segments of the protein that were exposed in the monomer but buried in the tetramer were identified. Out of those identified segments, TTR 89–97 was one that was highly buried (13% accessible) in the tetramer and freely accessible in the monomer. Rabbit polyclonal antibodies were raised against TTR 89–97 [13]. These antibodies were shown to bind TTR monomers and fibrils but not tetramers. From immunohistochemistry experiments, the antibody was shown to stain amyloid deposits in cardiac and other tissues from various hereditary and wild-type cases of ATTR, but not in pathological controls and healthy tissue. Notably, anti-TTR (89–97) was able to inhibit fibril formation at substoichiometric concentrations. Nanomolar amounts of antibody were sufficient to inhibit the fibrillation of micromolar amounts of TTR. Furthermore, these experiments utilized TTR protein, buffer, antibody, and no other components of the immune system. Thus, the fibril inhibition in this experiment is a result of fibril inhibitor activity of the antibody and not the immune system. Other experiments show that this antibody can activate the immune system. Immune complexes formed between the antibody and TTR fibrils were recognized and phagocytosed by human macrophages in culture [13]. Thus, this antibody may be one of those depleter antibodies that possess dual action in reducing fibril deposits, through fibril inhibitor action, and facilitate the immune clearance of amyloid fibrils and fibril intermediates. It was at this point that a collaboration with Prothena, a company from South San Francisco, USA, was initiated to develop and humanize monoclonals that are equivalent to the anti-TTR (89–97) polyclonal. The antigen that was used to generate rabbit polyclonals was also used to generate mouse monoclonals. The hybridoma screen revealed several monoclonal antibodies that possessed very similar properties to the rabbit polyclonal [14]. These antibodies specifically bind monomers and fibrils, but not tetramers. They specifically recognized TTR amyloid in the heart and other affected tissue. They inhibit the fibril formation of TTR, and they form immune complexes with misfolded TTR that activate the immune system [14]. One of these monoclonal antibodies was humanized and was designated PRX004.

The phase 1 clinical trial for PRX004 was started in 2018 but had to be terminated in 2020 due to the COVID-19 pandemic. Fortunately, the data gathered during that time were sufficient to aid decision making on whether to progress to a phase 2 clinical trial. PRX004 was shown to be safe and well tolerated at all doses examined. No serious adverse effects related to PRX004 administration were observed. The neuropathy impairment score (NIS) was used to evaluate muscle weakness, reflex loss, and sensory loss in the trial participants. All evaluable participants receiving treatment had NIS scores that were more favorable than published historical data, indicating that the rate of neuropathy decline is reduced with treatment. Some patients actually showed improvement in muscle strength, reflexes, and sensory perception. The cardiac function of the evaluable participants was assessed with the echocardiographic parameter of global longitudinal strain (GLS). These participants had improved GLS scores indicating improvement in cardiac health. Prothena has made this information available on their website (https://s201.q4cdn.com/351053094/files/doc_presentations/2021/04/1/AAN-PRX004-Ph1_20March21-FINAL.pdf, accessed on 15 September 2024). A phase 2 clinical trial sponsored by Novo Nordisk A/S., Copenhagen, Denmark was approved and is underway [15]. Coramitug is now the new designation for PRX004.

## 5. Cryo-EM Structure of the TTR Amyloid Fibril

Several techniques such as X-ray diffraction [51] and solid-state NMR [52] have been used to investigate the structures of TTR amyloid fibrils. While these techniques are capable of determining high-resolution structures of amyloid fibrils, cryo-EM is currently the only technique capable of producing 3D structures of single-patient-derived TTR amyloid fibrils. Here, we will only be discussing cryo-EM structures of patient-derived TTR fibrils.

**Figure 2:**
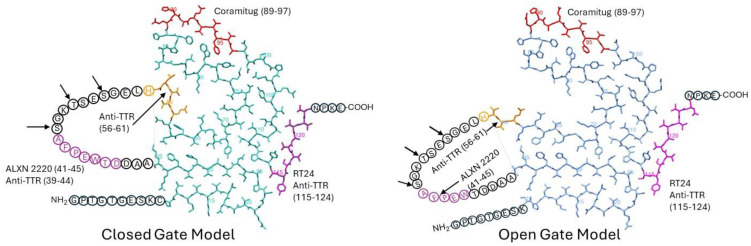
Top-down view of the closed-gate and open-gate model structures of TTR fibril subunits. The structures of TTR amyloid fibrils show variation between patients and within a single patient. A wild-type ATTR patient [53] shows a preference for the closed-gate model on the left; an I48S patient can adopt either a closed- or open-gate model [55]. In the above structures, amino acid residues represented as encircled one-letter codes are residues that are present in the sequence but not detectable in the cryo-EM structures. The arrows indicate some common protease cleavage sites [56]. The residue numbers specifying the location of the epitope are given in parentheses. The PDB codes for the closed- and open-gate models are 8ADE (in cyan) and 8TDO (in blue), respectively. Protein structures were rendered using Chimera 1.13.1.

The cryo-EM structure of amyloid fibrils from a patient with wild-type ATTR [53] is shown in Figure 1. For demonstration purposes, the fibrils are displayed as stacks of flattened monomers ranging from one to seven monomers per fibril. The natural lengths of TTR amyloid fibrils are between 60 and 220 monomers per fibril, as extrapolated from studies on the Alzheimer amyloid peptide [54]. Thus, the fibrils shown in Figure 1 are minute compared to natural fibrils. Each monomer is approximately 7 nm × 6 nm × 1 nm, and its polypeptide chain lies within this flat slab in an irregular conformation composed of b-strands connected by loops, which can be better appreciated in Figure 2, a top-down view of the flattened monomer.

The models start with the first 9 or 10 residues (in encircled text) being either unstructured in the wild type or missing in the I48S mutant (Figure 2). The fibril structure starts at residue 10. Residues 10–35 form a non-hydrogen bonded hairpin-like structure. At residue 36, the polypeptide chain leaves the fibril, forming an unstructured loop spanning residues 36–57 (in encircled text). The polypeptide chain re-enters the fibril at residue 58 (first residue after the loop ends), where it continues to form its irregular collection of b-strands and loops up to residue 123. It then leaves the fibril for four residues (in encircled text) and terminates at residue 127. The main difference between the open- and closed-gate models is in the conformation of residues 58–65, which, in turn, alters the position of the unstructured loop, residues 36–57.

The unstructured loop of residues 36–57 is also the site of proteolysis (Figure 2), a process which affects amyloid morphology [56]. Early studies have indicated a preference for proteolysis at residues 46, 49, and 52 [56]. However, recently solved cryo-EM structures of TTR fibrils also indicate a cleavage site at residue 58 [53,55]. The current data indicate considerable variability in the location of the cleavage sites, which may not yet be completely determined. After some consideration by the research community [57], the current opinion is that proteolysis occurs on-fibril rather than at the tetramer stage [53,55]. On-fibril proteolysis is likely a defense mechanism. Proteolyzed TTR likely dissociates from the fibril more easily than intact TTR, which would promote fibril disassembly. Similarly, proteolyzed TTR fragments are unlikely to reassemble and add on to the fibril; thus, they do not aid fibril growth. The role of loop proteolysis in ATTR is still being investigated.

## 6. Structural Disposition of Depleter Antibody Epitopes on Cryo-EM Structures of TTR Amyloid Fibrils

Earlier, we discussed five potential depleter antibodies that have been shown to stain amyloid deposits of TTR, and to bind to TTR fibrils but not bind to TTR tetramers. They are anti-TTR (39–44), anti-TTR (56–61), RT24, ALXN-2220, and Coramitug. In Figure 2, we indicate the locations of these epitopes on the closed- and open-gate models of TTR amyloid fibrils.

It is significant and satisfying to see that the potential depleter antibody epitopes all occupy solvent-exposed regions around the periphery of the flattened monomer subunit, because it means the epitopes would be accessible for binding by the antibody. It is noteworthy that these epitopes were selected before the cryo-EM structures of these fibrils were known. ALXN-2220, anti-TTR (39–44), and anti-TTR (56–61) epitopes were selected through screening tests, while Coramitug and RT24 were selected through hypothesis testing. This provides an example of how two different approaches led to the successful development of antibodies sharing similar important properties.

The anti-TTR (56–61) epitope is part of the unstructured loop spanning residues 36–57, and it contains the known proteolysis site at residue 58 [53,55]. In those patients where this proteolysis may happen, this epitope may either have a reduced availability or be completely lost. The ALXN-2220 epitope and anti-TTR (39–44) epitope are also part of the unstructured loop; however, they do not contain any known proteolysis sites. The position of the unstructured loop differs in the closed- versus open-gate models. Consequently, antibodies that target the loop, like anti-TTR (39–44) and ALXN-2220, may differ in their affinity for the closed- versus open-gate model structures. Coramitug and RT24 epitopes are part of the fibril structure, and the local structure around those epitopes does not differ greatly between the two models. Thus, Coramitug and RT24 may bind their respective epitopes in both the open- and closed-gate models with similar affinity.

Proteolysis generates short N-terminal fragments, 46–58 residues long, and longer C-terminal fragments, 69–81 residues long. The Coramitug epitope is located on the C-terminal fragment, while the ALXN-2220 epitope is located on the N-terminal fragment. Any differences in the effects of these antibodies may be attributable to which fragment is being bound by the antibody.

**Figure 3:**
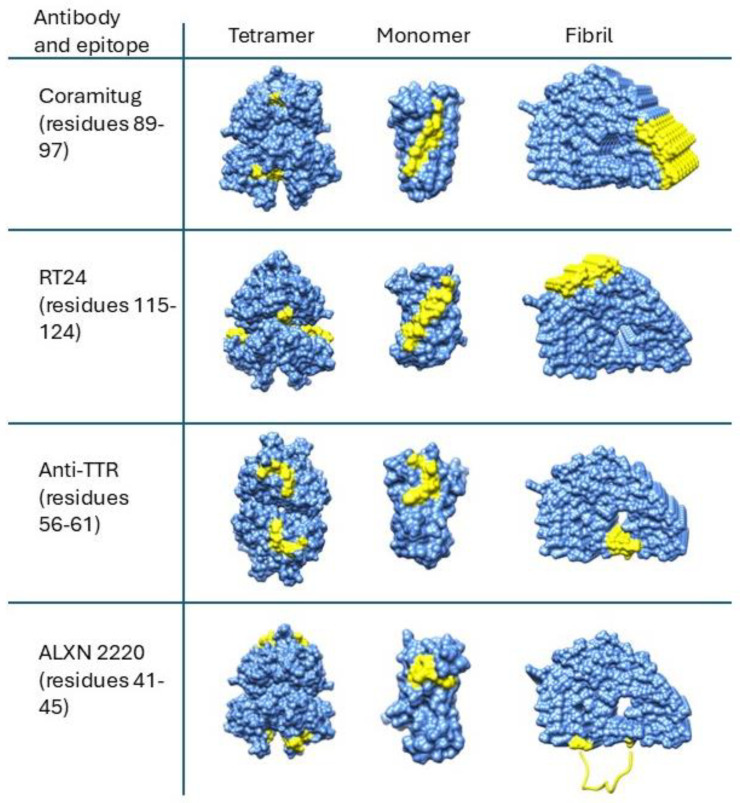
Structural disposition of potential depleter epitopes on TTR tetramers, monomers, and fibrils. Surface representation models of the TTR tetramer (3TCT), monomer (1GKO), and fibril (8ADE) are shown with the epitope residues in yellow and all other residues in blue. Regarding the fibril model showing the ALXN-2220 epitope in the bottom right corner of the figure, this epitope is part of the unstructured loop spanning residues 36–57 and is not visible in the cryo-EM structure. Instead, it is portrayed here as an unstructured loop (yellow). All the structures are oriented to maximize the view of the epitope. The fibril structures shown are derived from the closed-gate model (8ADE). The structural disposition of depleter epitopes on the open-gate model (8TDO) is qualitatively similar to those shown in the figure and is thus not shown here. Protein structures were rendered using Chimera 1.13.1.

Figure 3 shows a comparison of the accessibility of the epitopes on molecular surface representation models in the three structural states of the tetramer, monomer, and fibril.

Based on the fibril structures shown in Figure 3, all five epitopes appear accessible in the fibril state but display contextual differences. The epitopes of Coramitug and RT24 are part of the fibril structure and are likely to show dynamic movements that are similar to surface residues on globular proteins. The ALXN-2220 epitope, on the other hand, is part of the unstructured loop spanning residues 36–57. Consequently, the ALXN-2220 epitope is likely to be very flexible, which would aid antibody binding but might increase susceptibility to proteolysis. Fortunately, no protease sites are known to exist within the ALXN-2220 epitope. The anti-TTR (56–61) epitope is partly located within the central channel of the fibril structure, is less accessible compared to the others, and contains the proteolysis site at residue 58. These properties may potentially result in a reduced ability of this epitope to bind the antibody in the fibril.

In the monomer, all five depleter epitopes appear to be very accessible. Thus, all the antibodies should have similar utility in removing monomers by fibril inhibition and immune clearance, if tetramer binding is sufficiently low. In the tetramer, however, there is significant variation in the accessibility of the epitopes. The Coramitug epitope is highly buried in the tetramer, the RT24 epitope is slightly exposed, and the ALXN-2220 and anti-TTR (56–61) epitopes are slightly more exposed. Even weak binding to the tetramer can have important consequences for monoclonal therapy. Because native tetrameric TTR is present at the relatively high blood concentration of 3.6–7.2 µM (20 to 40 mg/dL) [58], it may sequester antibodies that show micromolar binding to the native TTR tetramer. In such cases, the native TTR tetramer serves to competitively bind the antibody, and consequently reduce the effective dose of antibody for therapy.

In conclusion, by examining the conformation of the depleter epitopes in the cryo-EM structures of the different TTR fibril models, we can better understand the properties of these antibodies. The anti-TTR (56–61) antibody appears to be the weakest of the group. It has the least accessible epitope on the fibril, and it contains a known proteolysis site at residue 58. Only RT24 and Coramitug were shown to inhibit TTR fibril formation, and the epitopes of these two antibodies also displayed the least accessibility in the native tetramer. For therapeutic purposes, the antibody needs to bind the monomer and the fibril, but not the tetramer. The Coramitug and RT24 epitopes are more deeply buried in the tetramer, and thus may result in minimal binding by the antibody. ALXN-2220 targets the loop spanning residues 36–57. The flexible nature of the ALXN-2220 epitope could facilitate antibody binding through induced fit into the antigen binding site. The epitopes of Coramitug and RT24 antibodies are rigid and likely bind with lock-and-key mechanisms.

**Figure 4:**
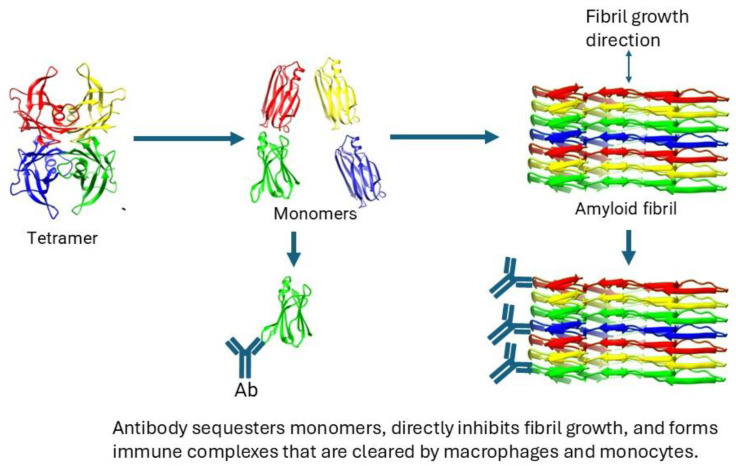
Schematic representation of the mechanism of antibody-mediated depletion of misfolded TTR in ATTR. Protein structures were rendered using Chimera 1.13.1.

The process to develop and understand TTR therapeutic antibodies by analyzing the structures of native and diseased forms of TTR is shown schematically in Figure 4. This process includes the analysis of the structures of the native state, the misfolding intermediates (like the monomer), and the amyloid fibril. The identification of epitopes differentially exposed between the native and diseased states has also been described. The ability to identify epitopes that are exposed in the fibril and fibril intermediates, but hidden in the native state, can potentially be used to develop future therapeutic antibodies for the many other systemic protein misfolding diseases [59].

## Figures and Tables

**Table 1:** Monoclonal antibody drugs in clinical trials for the treatment of ATTR, and related antibodies.

Antibody Name	Other Names	Epitope	Clinical Trial Numbers	Current Stage of Trial	Reference
ALXN-2220	NI006, NI301A, anti-TTR (41–45)	Residues 41–45	NCT06183931—Phase 3 NCT04622046—Phase 3—Japan	Phase 3	[10,11,12]
Coramitug	NNC6019-0001, PRX004, anti-TTR (89–97)	Residues 89–97	NCT03336580—Phase 1, terminated NCT05442047—Phase 2 NCT06260709—Phase 2—long term	Phase 2	[13,14,15]
RT24	anti-TTR (115–124)	Residues 115–124	-	-	[16]
anti-TTR (39–44)	-	Residues 39–44	-	-	[17,18,19,20,21]
anti-TTR (56–61)	-	Residues 56–61	-	-	[17]

**Table 2:** Binding affinities of some stabilizers and depleters.

	Affinities (K_d_) and Reference		
Drug	Tetramer	Monomer	Fibril
Thyroxine	Site 1: 3.2 nM; [39] Site 2: 8.1 µM; [39]	Not applicable	Not applicable
Flufenamic acid	Site 1: 30 nM; [40] Site 2: 255 nM; [40]	Not applicable	Not applicable
Tafamidis	Site 1: 5.1 nM; [41] Site 2: 203 nM; [41]	Not applicable	Not applicable
ALXN 2220	No binding detected	1.2 nM *; [11]	0.35 nM **; [11]
Coramitug	No binding detected	18.6 nM; [14]	0.62 µM **; [13]

* Note: The sample is a mixture of soluble low-order oligomers and monomers. ** Note: Due to technical difficulties, the affinity to fibrils is reported as an EC50 value.
